# Telomere length and incident atrial fibrillation – data of the PREVEND cohort

**DOI:** 10.1371/journal.pone.0171545

**Published:** 2017-02-03

**Authors:** Joylene E. Siland, Bastiaan Geelhoed, Isabelle C. van Gelder, Pim van der Harst, Michiel Rienstra

**Affiliations:** Department of Cardiology, University Medical Center Groningen, Groningen, the Netherlands; Medizinische Universitat Innsbruck, AUSTRIA

## Abstract

**Background:**

The incidence of atrial fibrillation (AF) increases with age. Telomere length is considered a marker of biological ageing. We investigated the association between leukocyte telomere length and incident AF in the Dutch Prevention of Renal and Vascular End-stage Disease (PREVEND) study.

**Methods:**

We included 7775 individuals without prevalent AF, and with leukocyte telomere length measured. Mean telomere length was determined by a monochrome multiplex quantitative polymerase chain reaction-based assay.

**Results:**

Mean age of our cohort was 49±13 years, and 50% were men. During a mean follow-up of 11.4±2.9 years incident AF was detected in 367 (4.7%) individuals. Telomere length was shorter in individuals developing incident AF compared to those without AF (p = 0.013). Incident AF was inversely related to the telomere length. In the quartile with the longest telomere length 68 (3.5%) individuals developed AF, in the shortest telomere length quartile 100 (5.1%) individuals (p = 0.032). Telomere length was associated with incident AF in the second shortest telomere length quartile using the longest telomere length quartile as reference (hazard ratio 1.64; 95% CI 1.02–2.66; p = 0.043). After including age or AF risk factors, the relation between telomere length and incident AF was no longer significant. We found a significant interaction of age, male sex, systolic blood pressure, BMI, heart failure, and myocardial infarction with telomere length for the association with incident AF.

**Conclusions:**

We found that shorter leukocyte telomere length is not independently associated with incident AF in a community-based cohort.

## Introduction

Advancing age is one of the major risk factors for atrial fibrillation (AF). The prevalence of AF increases with advancing age, to approximately 8% in those older than 80 years.[1–3] Approximately 70% of the individuals with AF are 65 to 80 years of age.[4,5] At all ages, AF is more frequently present in men than in women.[6–8] However, the exact reasons behind the influence of age on AF are not completely understood.

Telomere length shortens with advancing age and is considered to be a marker of biological aging.[9] Telomeres are DNA-protein complexes located at the ends of chromosomes, and are essential structures preventing DNA degradation. The ability of protection and maintenance of chromosomal stability becomes increasingly limited as a result of repeated cell division. When cell proliferation, senescence, or apoptosis occurs, this results in loss of telomere length.[10–12] Shortening of telomere length is observed in an inconsistent linear decline throughout life, and is considered to be a marker for biological aging.[9] Telomere length shortening is associated with cardiovascular diseases, including atherosclerosis, left ventricular hypertrophy and heart failure.[12]

Interestingly, AF is more common in men than in women, and men have shorter telomere lengths than women.[13] These observations may suggest a relation between telomere length and incident AF. However, in previous studies no relation has been found.[14] We hypothesize that shorter telomere length is associated with incident AF, as mechanism underlying the observed association between age and sex with incident AF. We studied the association of telomere length and incident AF in healthy individuals included in the community-based Dutch Prevention of Renal and Vascular End-stage Disease (PREVEND) cohort.

## Materials and methods

### Population

**Fig 1:**
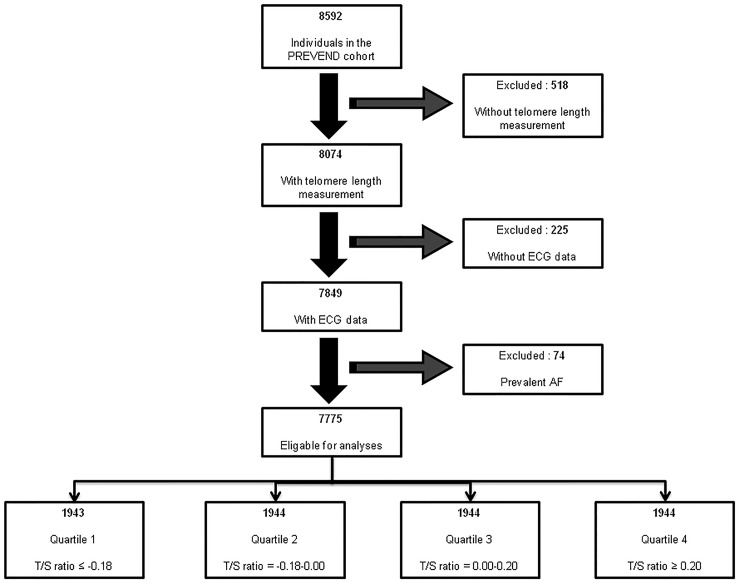
Exclusions for present study.

The association of telomere length and incident AF was examined in the PREVEND study, a community-based cohort, founded in 1997 in Groningen, the Netherlands. A detailed description of PREVEND has been published. In total, 8592 individuals were included in the PREVEND study. AF assessment has been described previously.[3] In brief, at each three-year interval study visit ECGs, blood and urine samples were collected. In addition, ECGs made at hospital visits and hospital admission between the study visits were screened for AF. We excluded individuals with no telomere length information (n = 518), individuals without ECG data (n = 225), and individuals with prevalent AF (n = 74) from analysis (Fig 1). The medical Ethics Committee of the University Medical Center Groningen approved the PREVEND study, and the study was conducted in accordance with the Declaration of Helsinki. All participants provided written informed consent.

### Covariate definitions

Systolic and diastolic blood pressure was calculated using an automatic Dinamap XL Model 9300 series device as the mean of the last two measurements of the two visits. Hypertension was defined as self-reported use of anti-hypertensive treatment, or as systolic blood pressure >140 mmHg, and diastolic blood pressure >90 mmHg. Antihypertensive treatment was defined as angiotensin receptor blockers, angiotensin converting enzyme inhibitors, calcium antagonists or diuretics. Body mass index (BMI) was defined as the ratio of weight to height squared (kg/m^2^), and obesity as a BMI >30 kg/m^2^. A fasting plasma glucose ≥7.0 mmol/L (126 mg/dL), or a non-fasting plasma glucose ≥11.1 mmol/L, or use of anti-diabetic treatment was appointed as diabetes mellitus. Total serum cholesterol of >6.5 mmol/L (251 mg/dl) or a serum cholesterol of >5.0 mmol/L (193 mg/dL) if a history of myocardial infarction was present or use of lipid lowering treatment was defined as hypercholesterolemia. Nicotine use in the last 5 years was described as smoking. Myocardial infarction or stroke was defined as participant-reported hospitalization for at least 3 days for this condition. Previously, an expert panel ascertained heart failure in detail.[15] The glomular filtration rate (eGFR) was measured using the simplified modification of diet formula. Measurements of highly sensitive C-reactive protein (highly sensitive CRP) and N-terminal prohormone of brain natriuretic peptide (NT-proBNP) were described previously.[16,17]

### Follow-up

Duration of follow-up was defined as the time between the baseline screening visit to first AF, the last contact date (end of the third PREVEND follow-up visit), or death. The latest last contact date was December 31, 2010.

### Leukocyte telomere length measurement

DNA was drawn from full blood using a standard DNA extraction kit (Qiamp, Qiagen, Venlo, the Netherlands). Mean leukocyte telomere length was determined from blood taken at the inclusion date using the monochrome multiplex quantitative polymerase chain method as described previously.[18,19] DNA samples were stored in a -80°C freezer, and no defrosting cycles were done before analyses. Methods of measurements, the reference gene and primer sequences have been described in detail before.[18] All DNA samples were assayed in triplicate. The samples were measured on different plates but in the same well position. In brief, 7 concentrations of a reference DNA sample of DNA concentrations were prepared by serial dilution and analyzed in triplicate in 384-well plates. As no-template control two wells received water, two wells received a human control sample, and, as a positive/max control, two wells were loaded with DNA of a human leukemia cell line with extreme long telomeres. The primers used for analyzes were; telomere, telg, ACACTAAGGTTTGGGTTTGGGTTTGGGTTTGGGTTAGTGT and telc, TGTTAGGTATCCCTATCCCTATCCCTATCCCTATCCCTAACA, to generate a short, fixed-length product. The S albumin primers were albu: CGGCGGCGGGCGGCGCGGGCTGGGCGGaaatgctgcacagaatccttg albd: GCCCGGCCCGCCGCGCCCGTCCCGCCGgaaaagcatggtcgcctgtt.

Finally, in the PCR consisted of the following concentrations: 1U Titanium Taq DNA polymerase with the provided Titanium Taq PCR buffer, 0.75xSYBR Green I (Sigma), 0.2 mM of each dNTP, 1 mM DTT, 1M betaine, 900nM of each telomere primers (Telg and Telc), 900nM of each albumin (Albu and Albd). The intra-assay coefficient of variation was 2.0% (T), 1.85% (S) and 4.5% (T/S ratio). Samples with a coefficient variation > 10% were re-run, when after re-run a coefficient of variation remained ≥ 10% the samples were omitted from statistical analyses.

### Statistical analyses

Characteristics of individuals are expressed as mean ± standard deviation, and median (interquartile range) for continuous variables and numbers (%) for categorical variables. For descriptive analysis, the fisher exact was used or Chi-square test for categorical data, the t-test or the Wilcoxon rank-test for continuous variables, depending on the normality of the data. For comparisons of more than two groups the Kruskal Wallis test or one way Anova test was used, depending on the normality of the data. A p-value of <0.05 was considered statistically significant for these tests. Leukocyte telomere length was expressed as a T/S, calculated by dividing the telomere (T) expression by the expression of a reference gene (S). Eventually, the leukocyte telomere length was calculated as (T/S ratio average—mean [T/S ratio average]) divided by standard deviation [T/S ratio average]. Since T/S ratios were skewed, ratios were logarithmically transformed and centered around 0. Logarithmically-transformed T/S ratio was discretized into quartiles. Time-to-event analyses using Cox proportional hazards models evaluate the association of T/S ratios and incident AF. In model 1, we performed unadjusted analyses, in model 2, we adjusted for age, in model 3 we adjusted for sex, in model 4 we adjusted for sex, BMI, antihypertensive treatment, myocardial infarction, stroke, heart failure, and PR-interval. As secondary analyses, Cox regression analyses were used to study the interactions between AF risk factors and telomere length for the association with incident AF (effect modification). Additionally, stratified Cox regression was performed for age categories and for men and women separately. A p-value of <0.05 was considered statistically significant.

## Results

### Individual characteristics

The 7775 included individuals had a mean age of 48.9±12.6 years, and 50% were men. During a mean follow-up of 11.4±2.9 years, 367 individuals developed incident AF (4.7%). Of the individuals with incident AF, 69% were men, and the mean age was 61.5±9.0 years (Table 1). Individuals that developed AF were older, more often men, and had more cardiovascular risk factors and diseases, except for smoking, which was less common in those with incident AF (39% versus 45%, p = 0.04). The most common cardiovascular risk factor in those with incident AF was hypertension (54%), obesity (25%), hypercholesterolemia (12%), and diabetes (10%).

**Table 1:** Baseline characteristics.

**Clinical profile**	**Total (n = 7775)**	**Incident AF (n = 367)**	**No AF (n = 7408)**	**P-value**
Telomere length	0.00 (-0.18–0.20)	-0.03 (-0.21–0.13)	0.00 (-0.18–0.21)	0.013
Age-years	49±13	62±9	48±12	<0.001
Male sex	3872 (50%)	254 (69%)	3618 (49%)	<0.001
BMI-kg/m2	25.6 (23.1–28.4)	27.5 (25.2–29.9)	25.5 (23.1–28.3)	<0.001
Obesity	1210 (16%)	90 (25%)	1120 (15%)	<0.001
Systolic blood pressure-mmHg	129±20	143±22	128±20	<0.001
Diastolic blood pressure-mmHg	74±10	79±9	74±10	<0.001
Heart rate-bpm	69±10	67±10	69±10	<0.001
Hypertension	2093 (27%)	198(54%)	1895 (26%)	<0.001
Heart failure	17 (0.2%)	6 (1.6%)	11 (0.1%)	0.002
Diabetes mellitus	285 (4%)	35 (10%)	250 (3%)	<0.001
Smoking	3442 (45%)	143 (39%)	3299 (45%)	0.040
Hypercholesterolemia	333 (4%)	42 (12%)	291 (4%)	<0.001
Glucose lowering treatment	107 (2%)	11 (3%)	96 (2%)	0.020
Lipid lowering treatment	314 (5%)	41 (13%)	273 (4%)	<0.001
Myocardial infarction	228 (3%)	50 (14%)	178 (2%)	<0.001
Stroke	54 (0.7%)	8 (2.2%)	46 (0.6%)	0.003
Antihypertensive treatment	1036 (16%)	131 (41%)	905 (15%)	<0.001
PR-interval-ms	158 (143–172)	168 (153–187)	158 (143–172)	<0.001
**Biomarker profile**	**Total (n = 7775)**	**Incident AF (n = 367)**	**No AF (n = 7408)**	**P-value**
eGFR-ml/min/1.73m2	80.9±14.5	76.6±15.7	81.1±14.4	<0.001
Creatinine-umol/L	82.0 (73.0–92.0)	88.0 (77.0–98.0)	82.0 (73.0–91.0)	<0.001
NTpro-BNP-ng/L	37.3 (16.6–72.6)	86.3 (40.8–195.5)	35.7 (16.0–69.0)	<0.001
hs-CRP -mg/L	1.27 (0.55–2.94)	1.97 (0.94–3.66)	1.25 (0.54–2.89)	<0.001
Glucose-mmol/L	4.9±1.2	5.4±1.7	4.9±1.2	<0.001

Data is expressed as mean ± standard deviation, median (interquartile range) or numbers (%). Telomere lengths are divided in quartiles. Abbreviations: AF = atrial fibrillation, BMI = body mass index, eGFR = estimate glomerular filtration rate, hs-CRP = highly sensitive C-reactive protein, NT pro-BNP = N-terminal prohormone of brain natriuretic peptide.

### Telomere length and risk of atrial fibrillation

**Fig 2:**
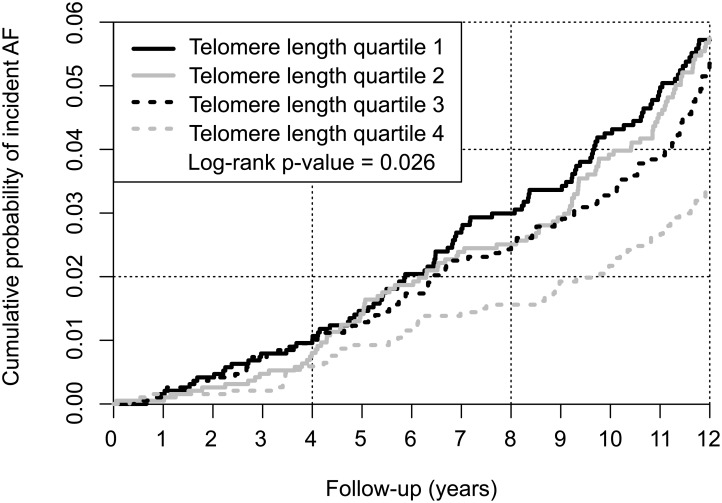
Cumulative incidence curve of incident AF, by quartiles of leukocyte telomere length. Quartile 1 represents the shortest telomere length group. Quartile 4 represents the longest telomere length group.

Telomere length was shorter in individuals developing incident AF compared to those without AF (p = 0.013). When discretizing individuals into quartiles based on the telomere length, the number of individuals with incident AF was inversely related to the telomere length. In the longest telomere length quartile 68 (3.5%) individuals developed incident AF, where 100 (5.1%) individuals developed AF in the shortest telomere length quartile (p = 0.032) (Fig 2, S1 Table). Telomere length was associated with incident AF in the second shortest telomere length quartile using the longest telomere length quartile as reference (hazard ratio 1.64; 95% confidence interval 1.02–2.66; p = 0.043). After age-adjusted analysis the relation between short telomere length and incident AF was no longer significant. In additional regression models, adjustments for sex and cardiovascular risk factors were performed, but in none of the models a significant association was found (Table 2). We performed secondary analyses with age as the time-variable, which led to similar findings as our primary analyses.

**Table 2:** Telomere length in quartiles and risk of incident AF.

	Telomere quartiles	HR(95% CI)	P-value
Unadjusted model	1. (≤ -0.18)	1.43(0.85–2.39)	0.181
	2. (-0.18–0.00)	1.64(1.02–2.66)	0.043
	3. (0.00–0.20)	1.32(0.83–2.09)	0.245
	4. (≥ 0.20)	1(reference)	
Age-adjusted model	1. (≤ -0.18)	0.69(0.44–1.10)	0.123
	2. (-0.18–0.00)	1.03(0.67–1.60)	0.882
	3. (0.00–0.20)	1.05(0.68–1.64)	0.820
	4. (≥ 0.20)	1(reference)	
Sex-adjusted model	1. (≤ -0.18)	1.38(0.82–2.32)	0.221
	2. (-0.18–0.00)	1.61(0.99–2.59)	0.053
	3. (0.00–0.20)	1.31(0.82–2.07)	0.258
	4. (≥ 0.20)	1(reference)	
Model adjusted for sex, BMI, antihypertensive treatment, myocardial infarction, stroke, heart failure, PR-interval	1. (≤ -0.18)	0.84(0.50–1.42)	0.517
	2. (-0.18–0.00)	1.19(0.72–1.96)	0.490
	3. (0.00–0.20)	1.19(0.71–1.98)	0.509
	4. (≥ 0.20)	1(reference)	

Hazard ratios are calculated for quartiles with shorter telomeres with the longest quartile as reference (quartile 4). The telomere length is the ratio of telomere expression divided by reference gene and is standardized per standard deviation. Log transformed T/S ratios were centered around 0. Abbreviations: AF = atrial fibrillation, BMI = body mass index, CI = confidence interval, HR = Hazard ratio.

### Effect modification of AF risk factors by telomere length

As secondary analyses, we studied effect modification of age, sex and other conventional AF risk factors. For most AF risk factors, we found a significant interaction with telomere length for the association with incident AF (S2 Table). For age, systolic blood pressure, BMI and heart failure the interaction with telomere length was >1, meaning that the presence of the risk factor weakens or counteracts the association between shorter telomere length and incident AF, or, equivalently that decrease in telomere length weakens or counteracts the association between incident AF and the risk factor. For male sex and stroke, the interaction with telomere length was <1, meaning that the presence of the risk factor strengthens the association between shorter telomere length and incident AF, or, equivalently that decrease in telomere length strengthens the association between incident AF and the risk factor. For myocardial infarction, and antihypertensive treatment no effect modification was found.

In addition, we performed stratified analyses in three age categories <50 years, 50–65 years, and >65 years. In the age category >65 years, the third shortest telomere length quartile was associated with AF (hazard ratio 2.70; 95% confidence interval 1.09–6.66, p-value = 0.031). No association between telomere length and incident AF was found in any of the other age categories or for one of the other telomere length quartiles (S3 Table).

In the analysis stratified for sex, the second shortest telomere length quartile in men was associated with AF (hazard ratio 2.06; 95% confidence interval 1.13–3.76, p-value = 0.018). No association between telomere length and incident AF was found for women (S4 Table).

## Discussion

One of the main risk factors of AF is advancing age. We therefore hypothesized an association between shorter telomere length and AF. However, the observed association between leukocyte telomere length and AF diminished when age was included in the model. Effect modification by most conventional risk factors was present, suggestive for a role of telomere length via AF risk factors in development of AF, although more research is needed.

### Telomere length, biological ageing and incident atrial fibrillation

Biological ageing is marked by shortening of telomere length, and short telomere length is associated with several age-related cardiovascular diseases, including atherosclerosis, left ventricular hypertrophy and heart failure.[12] Telomere length has been suggested to be susceptible to age-related diseases.[20] Our finding that telomere length is not independently associated with AF is consistent with an analysis of the Cardiovascular Health Study investigators, who also, found no association between telomere length and incident AF.[14] The Cardiovascular Health Study analysis was restricted to individuals >65 years in the United States, and sample size was modest (1639 individuals). We included 7775 individuals with a wide age range between 29 and 74 years. Nevertheless, results of both studies were consistent. Reasons for the absence of an independent relation between telomere length and incident AF can be the following. First, telomere length was measured in leukocytes, and this may not be fully representative of telomere length in atrial cells, [14] although several previous studies found a positive relation between telomere length in leukocytes and AF-related cardiovascular diseases.[14, 21–24] Differentiated cardiomyocytes undergo limited cell division after embryogenesis, and telomere length shortening in left atrial cardiomyocytes and dividing leukocytes may be different. However, telomere biology in left atrial cardiomyocytes is thought to play a role in the initiation of AF.[14] Premature apoptosis of cardiomyocytes resulting from loss of telomere length may contribute to fibrous replacement, and subsequently facilitate electrical chaotic activity (re-entry) that occurs in AF.[25–27] Secondly, other risk factors may contribute more than telomere length to the association of ageing and AF. Chronological ageing has been proven a risk factor of incident AF after adjustments for presence of cardiovascular risk factors and diseases, in numerous community-based cohorts.[3,14,28,29] With ageing stiffening of the left ventricle, and subsequent more diastolic dysfunction, atrial enlargement may occur, which may set the stage for AF.[30,31] Also sinus node dysfunction, and increase in premature atrial beats with ageing may be other mechanisms increasing the susceptibility for AF.[32–35] Thirdly, since we found modification by most conventional risk factors of the relation between telomere length and incident AF, it is possible that telomere length is not directly and independently related to development of AF, but via AF risk factors. Whether the presence of an AF risk factor itself influences the relation between telomere length and incident AF, or whether telomere length influences the relation between an AF risk factor and incident AF cannot be determined on present data. The further clarify the importance of effect modification of most AF risk factors by telomere length more studies are warranted, that compare patients with the same risk factors but different telomere lengths. Finally, other not-measured or subclinical risk factors and diseases potentially present in individuals at risk for AF may have influenced the association between ageing and development of AF (residual confounding).

### Strengths and limitations

Strengths of our analysis are the well-characterized large cohort, and the prospective design. The study also had several limitations. First, AF ascertainment is insensitive to asymptomatic paroxysms of AF, so asymptomatic AF may have been overlooked. And no information on temporal patterns of AF was available. Secondly, undetected age-related (cardiovascular) diseases may have influenced the results (residual confounding). Thirdly, telomere length was measured in leukocytes, and not in atrial cardiomyocytes. Fourthly, telomere length is measured at one time-point, and telomere length declines throughout life and the rate of telomere length attrition could affect the incidence of AF.[9] Fifthly, long term storage may had an impact on telomere length, but the time frame of data collection was relatively small in our study, thus large differences in telomere length between sampling dates seem unlikely. All DNA samples were stored at -80°C, and no defrosting cycles were done before analyses. Lastly, results cannot be extended to other ethnicities, telomere lengths are longer in Afro-Americans for example, since the majority of individuals included were of European ancestry.

### Future perspectives

Despite our study results, telomere biology may be of importance in the development of AF-risk factors and AF-associated cardiovascular outcomes, such as stroke, heart failure, death, and the progression of AF. Effect modification by AF-risk factors was present, suggestive for a role of telomere length via AF risk factors in development of AF, although more research is needed. Furthermore, in a cross-sectional analysis of patients with and without a history of AF included in the Intermountain Heart Collaborative Study differences were found in telomere lengths according to paroxysmal, persistent and permanent AF.[36] So, the progression of AF may be associated with a higher rate of shortening of telomeres. Further investigation is needed to study telomere biology in the setting of AF.

## Conclusions

Shorter leukocyte telomere length is not associated with incident AF in the community-based cohort of PREVEND. Effect modification by most conventional risk factors was present, suggestive for a role of telomere length via AF risk factors in development of AF, although more research is needed.

## Supporting information

S1 Table Baseline characteristics according to quartiles. Data is expressed as mean ± standard deviation, median (interquartile range) or numbers (%). Telomere lengths are divided in quartiles. Abbreviations: AF = atrial fibrillation, BMI = body mass index, eGFR = estimate glomerular filtration rate, hs-CRP = highly sensitive C-reactive protein, NT pro-BNP = N-terminal prohormone of brain natriuretic peptide.
(PDF)

S2 Table Association of telomere length and incident AF, modified by AF risk factor. ^**a**^ No significant interaction means that no effect modification was present for that AF risk factor. An interaction hazard ratio <1 means that the presence of the risk factor strengthens the association between shorter telomere length and incident AF, or, equivalently that a decrease in telomere length strengthens the association between incident AF and the risk factor. Similarly, an interaction hazard ratio >1 means that the presence of the risk factor weakens or counteracts the association between shorter telomere length and incident AF, or, equivalently that an increase in telomere length intensifies the association between incident AF and the risk factor. Abbreviation: BMI = body mass index. ^a^ Model includes AF risk factor, telomere length and the interaction-term of both. ^b^ All continues covariates were centered around their means.
(PDF)

S3 Table Cox regression models by age categories. Hazard ratios are calculated for quartiles with shorter telomeres with the longest quartile as reference. The telomere length is the ratio of telomere expression divided by reference gene and is standardized per standard deviation. Log transformed T/S ratios were centered around 0. Abbreviations: AF = atrial fibrillation, CI = confidence interval, HR = Hazard ratio.
(PDF)

S4 Table Cox regression models by sex. Hazard ratios are calculated for quartiles with shorter telomeres with the longest quartile as reference. The telomere length is the ratio of telomere expression divided by reference gene and is standardized per standard deviation. Log transformed T/S ratios were centered around 0. Abbreviations: AF = atrial fibrillation, CI = confidence interval, HR = Hazard ratio.
(PDF)
