# Supplementary material for: Telomere length and incident atrial fibrillation – data of the PREVEND cohort
Source: PLoS One. 2017 Feb 3;12(2):e0171545. doi: 10.1371/journal.pone.0171545 (PMC5291433; doi:10.1371/journal.pone.0171545)
Supplement: S1 Table — Data is expressed as mean ± standard deviation, median (interquartile range) or numbers (%). Telomere lengths are divided in quartiles. Abbreviations: AF = atrial fibrillation, BMI = body mass index, eGFR = estimate glomerular filtration rate, hs-CRP = highly sensitive C-reactive protein, NT pro-BNP = N-terminal prohormone of brain natriuretic peptide. (PDF) [file pone.0171545.s001.pdf]

Supplementary Table 1. Baseline characteristics according to quartiles.

Clinical profile	1st quartile (≤ -0.18) (n=1943)	2nd quartile (-0.18 - 0.00) (n=1944)	3rd quartile (0.00 - 0.20) (n=1944)	4th quartile (≥ 0.20) (n=1944)	P-value for trend
Age-years	52±13	49 ±12	48±12	46±12	<0.001
Male sex	1038 (53%)	1001 (51%)	942 (48%)	891 (46%)	<0.001
BMI-kg/m^2	26.3 (23.7-28.9)	25.6 (23.2-28.6)	25.4 (23.1-28.3)	25.1 (22.7-27.7)	<0.001
Obesity	361 (19%)	317 (16%)	299 (16%)	233 (12%)	<0.001
Systolic blood pressure-mmHg	133±21	129±20	128±20	126±19	<0.001
Diastolic blood pressure-mmHg	75±10	74±10	74±10	73±10	<0.001
Heart rate-bpm	70±10	69±10	69±10	69±10	0.002
Hypertension	658 (34%)	532 (27%)	496 (26%)	407 (21%)	<0.001
Heart failure	6 (0.3%)	5 (0.3%)	6 (0.3%)	0 (0.0%)	0.109
Diabetes mellitus	106(6%)	65 (3%)	68 (4%)	46 (2%)	<0.001
Smoking	911 (47%)	874 (45%)	839 (43%)	818 (42%)	0.018
Hypercholesterolemia	107 (6%)	103 (6%)	70 (4%)	53 (3%)	<0.001
Glucose lowering treatment	42 (3%)	24 (1%)	23 (1%)	18 (1%)	0.011

Lipid lowering treatment	110 (7%)	95 (6%)	58 (4%)	51 (3%)	<0.001
Myocardial infarction	85 (4.5%)	66 (3.5%)	48 (2.5%)	29 (1.5%)	<0.001
Stroke	16 (0.8%)	13 (0.7%)	15 (0.8%)	10 (0.5%)	0.649
Antihypertensive treatment	331 (20%)	301 (19%)	221 (14%)	183 (12%)	<0.001
PR-interval-ms	160 (147-173)	158 (143-173)	157 (143-172)	157 (143-170)	<0.001
**Biomarker profile**	**1st quartile (≤ -0.18) (n=1943)**	**2nd quartile (-0.18 - 0.00) (n=1944)**	**3rd quartile (0.00 - 0.20) (n=1944)**	**4th quartile (≥ 0.20) (n=1944)**	**P-value for trend**
eGFR-ml/min/1.73m^2	79.2±14.9	80.8±14.8	81.1±14.1	82.4±14.1	<0.001
Creatinine-umol/L	84.0 (75.0-93.0)	83.0 (74.0-92.0)	82.0 (73.0-91.0)	80.0 (73.0-90.0)	<0.001
NTpro-BNP-ng/L	40.1 (18.0-80.8)	36.5 (16.3-71.0)	38.5 (16.6-72.9)	34.2 (15.6-66.7)	<0.001
hs-CRP-mg/L	1.63 (0.72-3.58)	1.28 (0.56-3.06)	1.18 (0.54-2.73)	1.05 (0.48-2.49)	<0.001
Glucose-mmol/L	5.1±1.4	4.9±1.1	4.9±1.2	4.7±1.0	<0.001

Data is expressed as mean ± standard deviation, median (interquartile range) or numbers (%). Telomere lengths are divided in quartiles. Abbreviations: AF=atrial fibrillation, BMI=body mass index, eGFR=estimate glomerular filtration rate, hs-CRP=highly sensitive C-reactive protein, NT pro-BNP=N-terminal prohormone of brain natriuretic peptide.
