# Supplementary material for: Telomere length and incident atrial fibrillation – data of the PREVEND cohort
Source: PLoS One. 2017 Feb 3;12(2):e0171545. doi: 10.1371/journal.pone.0171545 (PMC5291433; doi:10.1371/journal.pone.0171545)
Supplement: S2 Table — a No significant interaction means that no effect modification was present for that AF risk factor. An interaction hazard ratio <1 means that the presence of the risk factor strengthens the association between shorter telomere length and incident AF, or, equivalently that a decrease in telomere length strengthens the association between incident AF and the risk factor. Similarly, an interaction hazard ratio >1 means that the presence of the risk factor weakens or counteracts the association between shorter telomere length and incident AF, or, equivalently that an increase in telomere length intensifies the association between incident AF and the risk factor. Abbreviation: BMI = body mass index. a Model includes AF risk factor, telomere length and the interaction-term of both. b All continues covariates were centered around their means. (PDF) [file pone.0171545.s002.pdf]

Supplementary Table 2. Association of telomere length and incident AF, modified by AF risk factor.[a]

Interaction term of telomere length and AF risk factors[b]	HR(CI)	P-value
Age * telomere length (per 10 increase)	1.86(1.09-3.18)	0.023
Male sex * telomere length	0.29(0.10-0.83)	0.021
Systolic blood pressure * telomere length (per 100 increase)	6.63(1.07-4.12×10^{1})	0.042
Antihypertensive treatment * telomere length (per 10 increase)	3.71(0.00-4.63×10^{5})	0.827
BMI * telomere length (per 10 increase)	2.11(1.06-4.19)	0.033
Heart failure * telomere length (per 0.1 increase)	1.94(1.17-3.21)	0.010
Myocardial infarction * telomere length (per 10 increase)	0.13(0.00-4.16×10^{7})	0.839
Stroke * telomere length (per 0.1 increase)	0.59(0.45-0.78)	<0.001

No significant interaction means that no effect modification was present for that AF risk factor. An interaction hazard ratio <1 means that the presence of the risk factor strengthens the association between shorter telomere length and incident AF, or, equivalently that a decrease in telomere length strengthens the association between incident AF and the risk factor. Similarly, an interaction hazard ratio >1 means that the presence of the risk factor weakens or counteracts the association between shorter telomere length and incident AF, or, equivalently that an increase in telomere length intensifies the association between incident AF and the risk factor. Abbreviation: BMI=body mass index.

[a] Model includes AF risk factor, telomere length and the interaction-term of both.

[b] All continues covariates were centered around their means.
