# Supplementary material for: Telomere length and incident atrial fibrillation – data of the PREVEND cohort
Source: PLoS One. 2017 Feb 3;12(2):e0171545. doi: 10.1371/journal.pone.0171545 (PMC5291433; doi:10.1371/journal.pone.0171545)
Supplement: S3 Table — Hazard ratios are calculated for quartiles with shorter telomeres with the longest quartile as reference. The telomere length is the ratio of telomere expression divided by reference gene and is standardized per standard deviation. Log transformed T/S ratios were centered around 0. Abbreviations: AF = atrial fibrillation, CI = confidence interval, HR = Hazard ratio. (PDF) [file pone.0171545.s003.pdf]

Supplementary Table 3. Cox regression models by age categories.

	**Telomere quartiles**	**HR(95% CI)**	**P-value**
Unadjusted model (individuals aged <50 years)	1. (≤ -0.18)	1.44(0.31-6.76)	0.645
	2. (-0.18-0.00)	1.29(0.37-4.47)	0.690
	3. (0.00-0.20)	0.95(0.28-3.26)	0.940
	4. (≥ 0.20)	1	
Unadjusted model (individuals aged 50-65 years)	1. (≤ -0.18)	0.66(0.34-1.29)	0.227
	2. (-0.18-0.00)	0.69(0.35-1.37)	0.294
	3. (0.00-0.20)	0.62(0.32-1.19)	0.148
	4. (≥ 0.20)	1	
Unadjusted model (individuals aged >65 years)	1. (≤ -0.18)	1.21(0.41-3.61)	0.727
	2. (-0.18-0.00)	2.52(0.94-6.74)	0.065
	3. (0.00-0.20)	2.70(1.09-6.66)	0.031
	4. (≥ 0.20)	1	

Hazard ratios are calculated for quartiles with shorter telomeres with the longest quartile as reference. The telomere length is the ratio of telomere expression divided by reference gene and is standardized per standard deviation. Log transformed T/S ratios were centered around 0. Abbreviations: AF=atrial fibrillation, CI=confidence interval, HR=Hazard ratio.
